# Clinical trial data transparency should match data access to scientific purpose: a tiered framework for reproducibility and reuse

**DOI:** 10.1186/s12916-026-05349-y

**Published:** 2026-10-22

**Authors:** Ulrich Mansmann, Ka Hin Tai, Giulia Varvarà, Florian Naudet

**Affiliations:** 1 IBE, LMU Munich, Munich, Germany; 2 Univ Rennes, CHU Rennes, Inserm, Institut de recherche en santé, environnement et travail (Irset)-UMR_S 1085, Rennes, 35000 France

**Keywords:** Clinical trial data sharing, Reproducibility, Data transparency, Publication-time reduced datasets, Controlled access, Data reuse

## Abstract

**Background:**

Clinical trial data sharing is increasingly endorsed by journals, funders, regulators, and research organisations. Nevertheless, access to individual participant data (IPD) remains inconsistent and may not be available when trial results are published. Access timelines can reflect several distinct components, including scientific and governance review, contractual and legal procedures, data preparation, de-identification, transfer, and subsequent harmonisation. When original data are unavailable, researchers may reconstruct approximate participant-level event-time data from published Kaplan-Meier curves. Trinquart and Stockler recently proposed that trialists instead release minimal analysable time-to-event data at publication. Their proposal raises a broader question: what kinds of data and documentation are required for different forms of reproducibility and scientific reuse?

**Main text:**

We argue that clinical trial data transparency should be considered in relation to the scientific claims and research activities that a particular data object can support. We propose a three-tier framework for data intentionally released or made accessible by trialists or data custodians. *Tier 1* comprises original publication-time reduced datasets and supports endpoint-level numerical reproducibility and analyses that are functions of the released observations. *Tier 2* comprises reproducibility packages containing analysis-ready data, code, derivation logic, and documentation sufficient to reproduce the reported analytic process. *Tier 3* provides governed access to broader de-identified IPD for substantively new analyses and reassessment of operational and analytic decisions. Reconstructed pseudo-IPD are considered separately because they are generated externally from publications rather than shared from the trial database. Privacy protection, governance, and processing environment are cross-cutting dimensions and do not map uniquely to a particular tier. They also apply differently to each tier.

**Conclusions:**

Publication-time reduced datasets can provide meaningful and timely transparency when broader IPD are not yet accessible. Their value should be recognised without confusing endpoint-level numerical reproducibility with reproduction of the complete analytic process or broader scientific reuse. We propose that publication-time reduced datasets should form a practical floor for timely transparency, while reproducibility packages and governed access to broader IPD provide progressively richer opportunities for reproducing, interrogating, and extending clinical trial analyses.

## Background

Clinical trial data sharing has been increasingly endorsed by journals, funders, regulators, and research organisations. Nevertheless, access to independent patient data (IPD) remains inconsistent and may require considerable time [1–5]. Importantly, such timelines can reflect several distinct components, including scientific and governance review, negotiation of data-use agreements, legal and contractual procedures, data preparation and de-identification, secure transfer, and subsequent harmonisation. It is therefore important to distinguish delays arising from the access governance process from those arising from operational, legal, or data-preparation requirements. When original data are unavailable, external researchers may reconstruct approximate participant-level event-time data from published Kaplan-Meier curves [6]. Such reconstruction demonstrates both the scientific demand for analysable data and the limitations of relying exclusively on published graphical information.

Trinquart and Stockler proposed that trialists release stripped-down datasets alongside reports of cancer randomised trials to reduce friction and facilitate publication-time transparency [7]. Without the intention to replace full IPD sharing, minimal analysable data underlying published time-to-event results could provide a timely complement to broader governed access.

This distinction is important. The relevant comparison is not between a reduced dataset and an ideal comprehensive IPD resource. It is between prompt availability of a scientifically useful reduced dataset and common alternatives at the time of publication: no analysable data, later access to broader IPD through governed processes, or approximate reconstruction from published information.

We argue that the current discussion of clinical trial data sharing would benefit from distinguishing data objects according to the scientific claims and research activities they can support. “Data sharing” is not a single activity with a single reproducibility objective. Reproducing a published numerical result, reproducing the analytic process that generated it, and using trial data to address substantively new questions require different combinations of data, documentation, code, and access arrangements.

We therefore propose a three-tier framework for data objects intentionally released or made accessible by trialists, sponsors, or data custodians. The terms “reconstructed pseudo-IPD”, “publication-time reduced dataset”, and the tier labels are used descriptively in this Debate and are not intended as established methodological categories.

## Main text

### Different scientific purposes require different data objects

Three related but distinct objectives should be separated.

*Endpoint-level numerical reproducibility* concerns the ability to reproduce results that are functions of released observations. Examples include reproducing Kaplan-Meier curves, event counts, unadjusted treatment-effect estimates, or alternative summaries of the observed event-time distribution.

*Analytic-process reproducibility* concerns recreating the published analytic workflow from analysis-ready data, derivation rules, statistical code, and accompanying documentation. This requires considerably more information than the observations underlying a particular figure or estimate.

*Broader scientific reuse* concerns analyses that extend beyond the published analytic framework. Such work may involve reconsidering endpoint construction, missing-data assumptions, estimand strategies, covariate definitions, treatment-effect heterogeneity, or other analytic and operational decisions.

These objectives require increasingly rich information, but they should not be interpreted as a simple hierarchy of scientific worth. A small, well-designed dataset released at publication may make an important contribution even when it cannot support reproduction of the complete analytic process or broader secondary research.

### Tier 1: publication-time reduced dataset

Tier 1 comprises an original reduced dataset released by trialists at or near publication. For a time-to-event analysis, it may contain per patient his/her treatment assignment, observed event or censoring time, event indicator, and a limited number of additional variables.

A Tier 1 time-to-event dataset can support reproduction of Kaplan-Meier curves, numbers of events and censored observations, log-rank tests, unadjusted Cox model estimates, milestone survival, and restricted mean survival time (RMST). It may also permit flexible survival modelling, investigation of non-proportional hazards, time-varying effect displays, reverse Kaplan-Meier estimation, comparison of censoring distributions between treatment groups, and event-time IPD meta-analysis.

These are meaningful forms of reproducibility and reuse. A reduced dataset should therefore not be dismissed merely because it cannot reproduce every aspect of the original trial analysis.

Its limitations concern what is not represented. Unless the necessary variables and documentation are included, a Tier 1 dataset generally cannot establish how endpoints were derived from source information, reproduce adjusted analyses, distinguish all reasons for censoring, reassess missing-data decisions, or redefine an estimand that requires different handling of treatment switching, new therapy, withdrawal, death, or other intercurrent events.

The concept of a publication-time reduced dataset is not restricted to time-to-event outcomes. The same principle can be applied to other trial endpoints whenever a limited set of participant-level observations is sufficient to reproduce the principal reported result or to support closely related analyses. Depending on the outcome, a Tier 1 dataset might therefore contain treatment assignment together with a continuous, binary, count, ordinal, or repeated-measures outcome and, where necessary, a small number of additional variables required to define the reported analysis. The specific content of a Tier 1 dataset should thus be determined by the endpoint and the inferential target rather than by a particular statistical model or outcome type.

A Tier 1 dataset may thus permit alternative summaries and models of the observed event-time distribution without allowing investigators to redefine the underlying endpoint or estimand. These inferential boundaries should be made explicit in a scope statement accompanying the dataset.

### Tier 2: reproducibility package

Tier 2 supports reproduction of the reported analytic process. It includes the analysis-ready dataset together with the statistical analysis plan, data dictionary, derivation logic, estimand definitions, analytic code, software information, and sufficient provenance to recreate the published analyses. The distinction from Tier 1 is therefore not that Tier 1 lacks reproducibility value. Rather, the two tiers address different reproducibility targets. Tier 1 enables reproduction and extension of analyses that are functions of released observations; Tier 2 seeks to reproduce the process by which the reported analyses were generated.

A Tier 2 package can make transparent how analysis populations, derived variables, censoring indicators, covariate adjustments, missing-data procedures, and sensitivity analyses were implemented. Recent case studies suggest that such packages are feasible and can facilitate post-publication scrutiny and reuse [8, 9].

Even a comprehensive reproducibility package has boundaries. It may remain insufficient for research questions requiring variables, source information, or operational documentation that were not incorporated into the analysis-ready dataset.

For example, consider a randomised trial with a continuous outcome measured repeatedly over follow-up and analysed using a mixed-effects model. A Tier 1 dataset might provide treatment assignment, visit time, and the observed outcome values, allowing alternative summaries of the observed longitudinal data. A Tier 2 reproducibility package would additionally provide the analysis-ready dataset, the rules used to define visits and derive change-from-baseline variables, the specification of fixed and random effects, the covariance structure, the handling of missing observations and protocol deviations, the prespecified contrasts used to estimate treatment effects, and the statistical code used for the primary and sensitivity analyses. Such a package would permit an independent researcher to reproduce not only the numerical result, but the analytic process that generated it.

### Tier 3: governed access to broader IPD

Tier 3 provides governed access to broader de-identified IPD and associated documentation [10]. It is intended for research that goes beyond the published analytic framework, including reassessment of derived variables, investigation of treatment-effect heterogeneity, incorporation of richer covariates, application of different missing-data assumptions, reconsideration of endpoint definitions, and evaluation of alternative estimand strategies.

The restoration of Study 329 illustrates why some questions require detailed participant-level information and supporting records that cannot be represented adequately in a publication-time reduced dataset alone [11].

Tier 3 does not necessarily imply unrestricted access to every source variable. Data provided may be curated to the approved research question, with proportionate scientific review, contractual safeguards, secure processing, and output checking.

Nor should Tier 3 be characterised by delay. Its defining characteristic is the capacity to address broader scientific questions and revisit analytic or operational decisions. The time required to obtain access may instead reflect several separable processes, including application review, contracting, legal requirements, data curation, de-identification, preparation, and secure transfer. Improving the predictability and efficiency of these processes is compatible with maintaining appropriate governance.

The tiers should not be interpreted as a simple cumulative hierarchy in which a higher tier necessarily subsumes all functions of the tiers below. Broader IPD made available through Tier 3 may be sufficient for many endpoint-level analyses, but without the exact analysis-ready dataset, derivation logic, and code used for the publication they may not permit reproduction of the complete reported analytic process. Conversely, providing broader data than required for a specific reproducibility objective may impose unnecessary preparation, access, and disclosure-control requirements. The principle underlying the framework is therefore proportionality: the data object, documentation, and access arrangements should be matched to the scientific purpose. Publication-time reduced datasets and reproducibility packages remain useful even when governed access to broader IPD is available, because they can provide a more direct and purpose-specific route to numerical or analytic-process reproducibility. Controlled-access platforms commonly review requests in relation to a prespecified research question and may tailor the data made available accordingly, further supporting the principle that access should be sufficient for the intended purpose rather than maximally inclusive. Increasing journal requirements for publication-time availability of the data and, in some cases, analytic code underlying reported results illustrate the practical value of purpose-specific Tier 1 and Tier 2 transparency packages alongside broader IPD-sharing mechanisms.

### Reconstructed pseudo-IPD as an external fallback

Reconstructed pseudo-IPD are generated by external researchers from published aggregate information, most commonly digitised Kaplan-Meier curves [7]. They do not originate from the trial database and should therefore be distinguished from original reduced datasets released by trialists.

Reconstruction may reproduce published survival probabilities, medians, and treatment-effect estimates with useful accuracy and can enable evidence synthesis when original data are unavailable. Its fidelity nevertheless depends on the resolution and completeness of published curves, numbers at risk, event counts, and reconstruction assumptions. The resulting data generally lack participant-level covariates, endpoint derivation information, reasons for censoring, and operational metadata.

A recent meta-analysis in patients with resectable non-small cell lung cancer illustrates both the value and the limitations of this approach [12]. By reconstructing study identifier, treatment group, event time, and event status across eight trials, the investigators enabled event-time evidence synthesis in the absence of original IPD. Such a dataset can support important survival analyses, but it should not be equated with an IPD meta-analysis based on original trial datasets containing richer covariates, derivations, and provenance [13].

Reconstruction is not limited to time-to-event outcomes. For binary or other discrete endpoints, sufficiently detailed published treatment-by-outcome counts can be expanded into participant-level rows that exactly reproduce the reported contingency table. This is particularly relevant for meta-analyses of binary outcomes, where publications often report the complete event and non-event counts by treatment group and study, so that the aggregate data required for the meta-analysis can be fully recovered even though no additional participant-level information is available.

Reconstructed pseudo-IPD should therefore be viewed as an important external fallback when original data are unavailable rather than as a substitute for intentional data sharing by trialists.

### Privacy protection, governance, and processing environments are cross-cutting dimensions

The proposed tiers classify data objects according to scientific content, documentation, and intended use. They do not uniquely determine whether data should be downloadable or analysed within a secure processing environment (SPE).

Disclosure risk depends on granularity, rarity, linkability, population context, and information available elsewhere, rather than simply on the number of variables contained in a dataset [8].

A publication-time reduced dataset may be released as an anonymised downloadable file when residual disclosure risk is sufficiently low, but an SPE may be appropriate when even a small dataset remains sensitive. The same principle applies to a Tier 2 reproducibility package: it may be downloadable after adequate anonymisation or accessed through an SPE if reproducing the analysis requires more detailed or linkable information [9].

Tier 3 will commonly require controlled access and secure processing because the information available is richer, but safeguards should remain proportionate to the assessed risk.

Anonymisation or de-identification, governance procedures, contractual conditions, and technical processing environments should therefore be reported as distinct dimensions rather than combined within a single tier label.

### Synthetic trial data as a separate, cross-cutting data object

Synthetic datasets do not fit naturally into the three tiers because they differ from original trial data in kind rather than only in completeness. Their suitability depends on the synthesis method, preservation of relationships relevant to the intended analysis, risks of attribution or membership disclosure, and empirical validation against the real data [14, 15].

Synthetic data may accompany any tier. They may be useful for teaching, demonstrating analytic code, developing workflows, or exploring data structures before governed access to real data is granted. They should not, however, be assumed to reproduce a trial result or guarantee privacy merely because they are labelled synthetic.

Claims concerning their analytic utility and disclosure protection should therefore be task-specific and empirically documented. Synthetic data are best described alongside the proposed tier framework rather than incorporated within it.

Synthetic data may also have a role in trial design and analysis by supplementing a concurrently randomised control group or, in selected settings, by contributing to an external or hybrid control arm. Such approaches aim to increase precision or reduce the number of participants who need to be randomised to control. However, their validity depends critically on the quality and relevance of the source data, the method used to generate or select the additional controls, comparability with the concurrent trial population, and appropriate evaluation of resulting bias and uncertainty. Synthetic controls should therefore be regarded as an analytical use of synthetic or external information rather than as a separate level within the proposed tier hierarchy.

### Operationalising the framework

The framework is intended as an initial operational proposal rather than a finished classification system. Table 1 separates purpose, data content, documentation, legitimate uses, limitations, disclosure control, processing environment, and governance. This avoids treating secure processing environments, anonymisation, scope statements, and governance procedures as interchangeable safeguards. Several practical guardrails could support implementation.

**Table 1:** Operational characteristics and scientific scope of the proposed three-tier framework

Aspect	Tier 1: Publication-time reduced dataset	Tier 2: Reproducibility package	Tier 3: Governed broader IPD access
Primary purpose	Endpoint-level numerical reproducibility and limited extension of analyses based on the released observations	Reproduction of the reported analytic process	Broader approved secondary research and reassessment of analytic or operational decisions
Typical content	Original reduced participant-level observations relevant to the reported endpoint, e.g. treatment and continuous, binary, ordinal, count, longitudinal, or time-to-event outcomes	Analysis-ready data, code, derivation logic, SAP, estimand definitions, and software information	Richer de-identified IPD and supporting documentation relevant to the approved question
Minimum documentation	Source report, variable and endpoint definitions, scope statement, and inferential limits, inferential target	Protocol, data dictionary, derivation rules, SAP, code, provenance, and instructions for execution	Protocol, data dictionary, SAP, curation record, metadata, governance documentation, and access conditions
Legitimate uses	Reproduction of principal endpoint results and closely related analyses determined by the released observations; for time-to-event data, e.g. Kaplan-Meier, log-rank, Cox, restricted mean survival time (RMST), censoring diagnostics, and event-time meta-analysis	Reproduction of published primary, secondary, and sensitivity analyses; examination of analytic implementation	Alternative derivations and estimand strategies; richer subgroup, missing-data, and heterogeneity analyses; broader IPD meta-analysis
Main limitation	Cannot usually verify source-level endpoint construction, omitted variables, all censoring reasons, or operational handling of intercurrent events	Limited to the variables and source information included in the package	Greater disclosure risk and more extensive curation, access review, and operational requirements
Disclosure control	Data minimisation and contextual disclosure-risk assessment; anonymisation where downloadable	Anonymisation or controlled access according to granularity and linkability	De-identification plus proportionate controlled access and output review
Processing environment	Downloadable file when adequately anonymised; SPE when residual risk requires it	Downloadable or SPE-based, depending on the information needed for reproduction	Usually, an SPE or another governed research environment
Governance	Light and proportionate to risk, with clear conditions of use	Proportionate to residual risk and access model	Proportionate formal application, review, agreement, monitoring, and, where appropriate, output checking

Tier 1 datasets should include clear variable and endpoint definitions, a statement of scope, and explicit description of inferential limits. Researchers should be able to determine not only what the released data permit them to reproduce, but also which elements of the original trial analysis cannot be reconstructed from the available information.

Tier 2 packages should provide sufficient data, code, software information, derivation rules, and documentation to reproduce the analyses for which reproducibility is claimed.

Tier 3 access should combine proportionate review with standardised agreements, transparent requirements, and predictable procedures. Where access is not immediate, reporting the separate contributions of review, contracting, legal processes, data preparation, and other operational steps would also help identify where avoidable delays occur.

SPIRIT 2025 and CONSORT 2025 reinforce expectations that trial protocols and reports describe whether, when, and how trial data and associated materials will be made available [16, 17]. The proposed framework could make such statements more informative by requiring investigators to specify not simply whether “data will be shared”, but what type of data object will be available, with what documentation, under what access conditions, and for what legitimate scientific purposes.

The framework may also provide a basis for empirical work. Existing data-sharing statements and shared data objects could be classified according to these dimensions to examine feasibility, identify borderline cases, and evaluate whether more informative transparency indicators or badge systems could be developed [18].

Most importantly, the framework avoids treating transparency as binary. A publication-time reduced dataset, a reproducibility package, and governed access to broader IPD are not competing implementations of the same concept. They address different scientific needs and can coexist within a coherent transparency strategy.

### Conclusions

Publication-time reduced datasets should be evaluated against realistic alternatives available when trial results are published: no analysable data, later access to broader IPD, or approximate reconstruction from published information. Against these comparators, original reduced datasets can represent a substantial improvement. They may provide meaningful endpoint-level numerical reproducibility and support alternative analyses of the observed data.

Their inferential limits nevertheless matter. Reproducing an event-time result differs from reproducing the complete analytic process, and both differ from conducting broader secondary research or revisiting how endpoints and estimands were constructed.

Reconstructed pseudo-IPD, publication-time reduced datasets, reproducibility packages, and governed access to broader IPD should therefore not be collapsed into a single notion of “data sharing”. They are different data objects serving different scientific purposes.

A useful transparency framework should match data content, documentation, privacy protection, processing environment, and governance arrangements to the claims and research activities that the available information can support.

We propose that publication-time reduced datasets should become a practical floor for timely transparency, not a ceiling for reproducibility and responsible reuse. Reproducibility packages and governed access to broader IPD can complement this foundation by enabling increasingly detailed reproduction, interrogation, and extension of clinical trial analyses.

## Data Availability

No datasets were generated or analysed during the current study.

## Abbreviations

CONSORT: Consolidated Standards of Reporting Trials
IPD: Individual participant data
NSCLC: Non-small cell lung cancer
RMST: Restricted Mean Survival Time
SAP: Statistical analysis plan
SPE: Secure processing environment
SPIRIT: Standard Protocol Items: Recommendations for Interventional Trials

## References

[1] Taichman DB, Sahni P, Pinborg A, et al. Data sharing statements for clinical trials: a requirement of the International Committee of Medical Journal Editors. Ann Intern Med. 2017;167:63–5. 10.2471/BLT.17.196733. 28586790

[2] Ohmann C, Banzi R, Canham S, et al. Status, use and impact of sharing individual participant data from clinical trials: a scoping review. BMJ Open. 2021;11:e049228. 10.1136/bmjopen-2021-049228. PMC8375721 34408052

[3] Scutt P, Woodhouse LJ, Montgomery AA, et al. Data sharing: experience of accessing individual patient data from completed randomised controlled trials in vascular and cognitive medicine. BMJ Open. 2020;10:e038765. 10.1136/bmjopen-2020-038765. PMC7482449 32912955

[4] Ventresca M, Schünemann HJ, Macbeth F, et al. Obtaining and managing data sets for individual participant data meta-analysis: scoping review and practical guide. BMC Med Res Methodol. 2020;20:113. 10.1186/s12874-020-00964-6. PMC7218569 32398016

[5] Tai KH, Varvarà G, Escoffier E, Mansmann U, DeVito NJ, Armond ACV, Naudet F. Data sharing policies, requirements, and support from public and private clinical trial sponsors: a survey on top sponsors of clinical trials in Europe. J Clin Epidemiol. 2026;199:112452. 10.1016/j.jclinepi.2026.112452. 42575483

[6] Guyot P, Ades AE, Ouwens MJNM, Welton NJ. Enhanced secondary analysis of survival data: reconstructing the data from published Kaplan-Meier survival curves. BMC Med Res Methodol. 2012;12:9. 10.1186/1471-2288-12-9. PMC3313891 22297116

[7] Trinquart L, Stockler MR. The paradox of data sharing in cancer randomized clinical trials: a call for greater transparency. JAMA Oncol. 2025;11:957–8. 10.1001/jamaoncol.2025.1984. 40608301

[8] Rodriguez A, Tuck C, Dozier MF, et al. Current recommendations and practices for anonymising data from clinical trials in order to make it available for sharing: a scoping review. Clin Trials. 2022;19:452–63. 10.1177/17407745221087469. PMC9373195 35730910

[9] Burgwinkel C, Chiam HC, Tai KH, et al. Supporting reanalysis and reuse of clinical trial data: a case study. Trials. 2026;27:351. 10.1186/s13063-026-09646-y. PMC13137473 41882735

[10] Sydes MR, Johnson AL, Knox C, et al. Sharing data from clinical trials: the rationale for a controlled access approach. Trials. 2015;16:104. 10.1186/s13063-015-0604-6. PMC4369803 25872927

[11] Le Noury J, Nardo JM, Healy D, et al. Restoring Study 329: efficacy and harms of paroxetine and imipramine in treatment of major depression in adolescence. BMJ. 2015;351:h4320. 10.1136/bmj.h4320. PMC4572084 26376805

[12] Marinelli D, Nuccio A, Federico AD, et al. Improved event-free survival after complete or major pathologic response in patients with resectable NSCLC treated with neoadjuvant chemoimmunotherapy regardless of adjuvant treatment: a systematic review and individual patient data meta-analysis. J Thorac Oncol. 2025;20:285–95. 10.1016/j.jtho.2024.09.1443. 39389220

[13] Riley RD, Lambert PC, Abo-Zaid G. Meta-analysis of individual participant data: rationale, conduct, and reporting. BMJ. 2010;340:c221. 10.1136/bmj.c221. 20139215

[14] Azizi Z, Zheng C, Mosquera L, et al. Can synthetic data be a proxy for real clinical trial data? A validation study. BMJ Open. 2021;11:e043497. 10.1136/bmjopen-2020-043497. PMC8055130 33863713

[15] El Kababji S, et al. Evaluating the utility and privacy of synthetic breast cancer clinical trial data sets. JCO Clin Cancer Inf. 2023;7:e2300116. 10.1136/bmjopen-2020-043497. PMC10703127 38011617

[16] Chan AW, Boutron I, Hopewell S, et al. SPIRIT 2025 statement: updated guideline for protocols of randomised trials. BMJ. 2025;389:e081477. 10.1136/bmj-2024-081477. PMC12035670 40294953

[17] Hopewell S, Chan AW, Collins GS, et al. CONSORT 2025 statement: updated guideline for reporting randomised trials. BMJ. 2025;389:e081123. 10.1136/bmj-2024-081123. PMC11995449 40228833

[18] Center for Open Science. Open Science Badges. https://www.cos.io/initiatives/badges Accessed 16 July 2026.

